# If exercise is medicine, where is the dose? A call to improve reporting and monitoring of exercise interventions in fibromyalgia research

**DOI:** 10.3389/fspor.2026.1777261

**Published:** 2026-04-17

**Authors:** Blanca Gavilán-Carrera, Álvaro-José Rodríguez-Domínguez, José-Carlos Tortosa-González, Manuel Delgado-Fernández

**Affiliations:** 1 PA-HELP “Physical Activity for Health Promotion, CTS-1018” Research Group, Department of Physical Education and Sports, Faculty of Sports Science, University of Granada, Granada, Spain; 2 Sport and Health University Research Institute (IMUDS), University of Granada, Granada, Spain; 3 Centro Universitario San Isidoro, Affiliated with Pablo de Olavide University, Seville, Spain

**Keywords:** aerobic exercise, exercise, exercise is medicine, exercise monitoring, exercise reporting, fibromyalgia, mind-body exercise, muscular strenght

## 1 Introduction

Exercise is widely recognized as a cornerstone non-pharmacological therapy for managing pain and other core symptoms of fibromyalgia. Current guidelines, including those from the American College of Sports Medicine (ACSM), frame exercise prescription through the FITT principles—frequency, intensity, time, and type—providing a structured approach to defining exercise dose in this population (1). When these parameters are implemented with high compliance (i.e., when at least 70% of the ACSM-recommended exercise dose is met), exercise interventions are associated with greater improvements in pain, sleep quality, and fatigue, as supported by recent meta-analytic evidence in fibromyalgia (2). However, only about half of the analyzed exercise interventions achieved high compliance with ACSM guidelines, highlighting substantial variability in exercise prescription across fibromyalgia trials (2).

**Figure 1:**
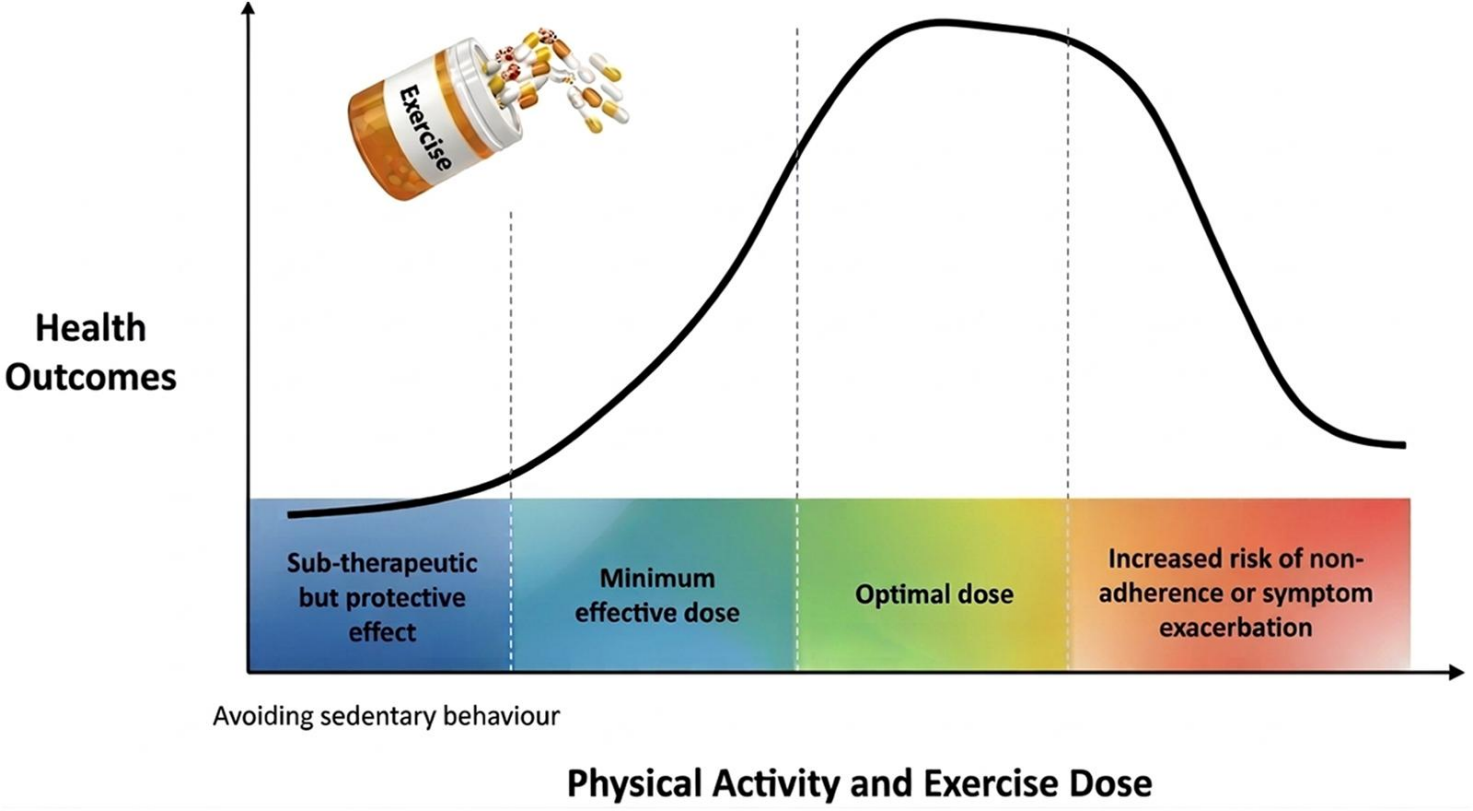
Conceptual dose–response relationship for physical activity and exercise in fibromyalgia. Physical activity dose is shown as a continuum, from protective low doses to a minimal effective dose and higher outcome-specific doses, beyond which additional benefits may plateau and tolerance or adherence may decline.

Despite this growing body of evidence, two complementary perspectives continue to coexist in both research and clinical practice. On one hand, public health recommendations emphasize that any physical activity is preferable to none, particularly in populations with a high symptom burden and low activity levels (3, 4). On the other hand, research increasingly seeks to identify specific exercise doses, operationalized through FITT parameters, that maximize therapeutic benefit. We argue that these perspectives do not represent opposing views, but rather reflect different positions along a dose–response continuum (Figure 1).

This continuum ranges from reducing the detrimental effects of sedentary behavior in individuals with fibromyalgia (5), to achieving a minimum effective dose for meaningful clinical improvement, and ultimately to defining outcome-specific optimal exercise doses (6). Within this framework, the message “move more and sit less” provides a simple, evidence-based recommendation easily understood by most patients; however, in clinical practice it must be translated into individualized exercise prescriptions to maximize benefits (7).

Recent meta-analyses provide important insights into the exercise dose–response relationship in fibromyalgia, particularly regarding pain reduction and overall disease impact (6, 8–13). While this literature reflects progress in FITT-based exercise prescription, dose–response interpretation remains limited by persistent methodological shortcomings, including heterogeneous interventions, incomplete reporting of FITT parameters, limited monitoring of performed exercise, high dropout rates, and poor adherence documentation. Consequently, conclusions often rely on intended or reported FITT criteria (when available) rather than on the exercise dose actually delivered and executed by participants.

**Figure 2:**
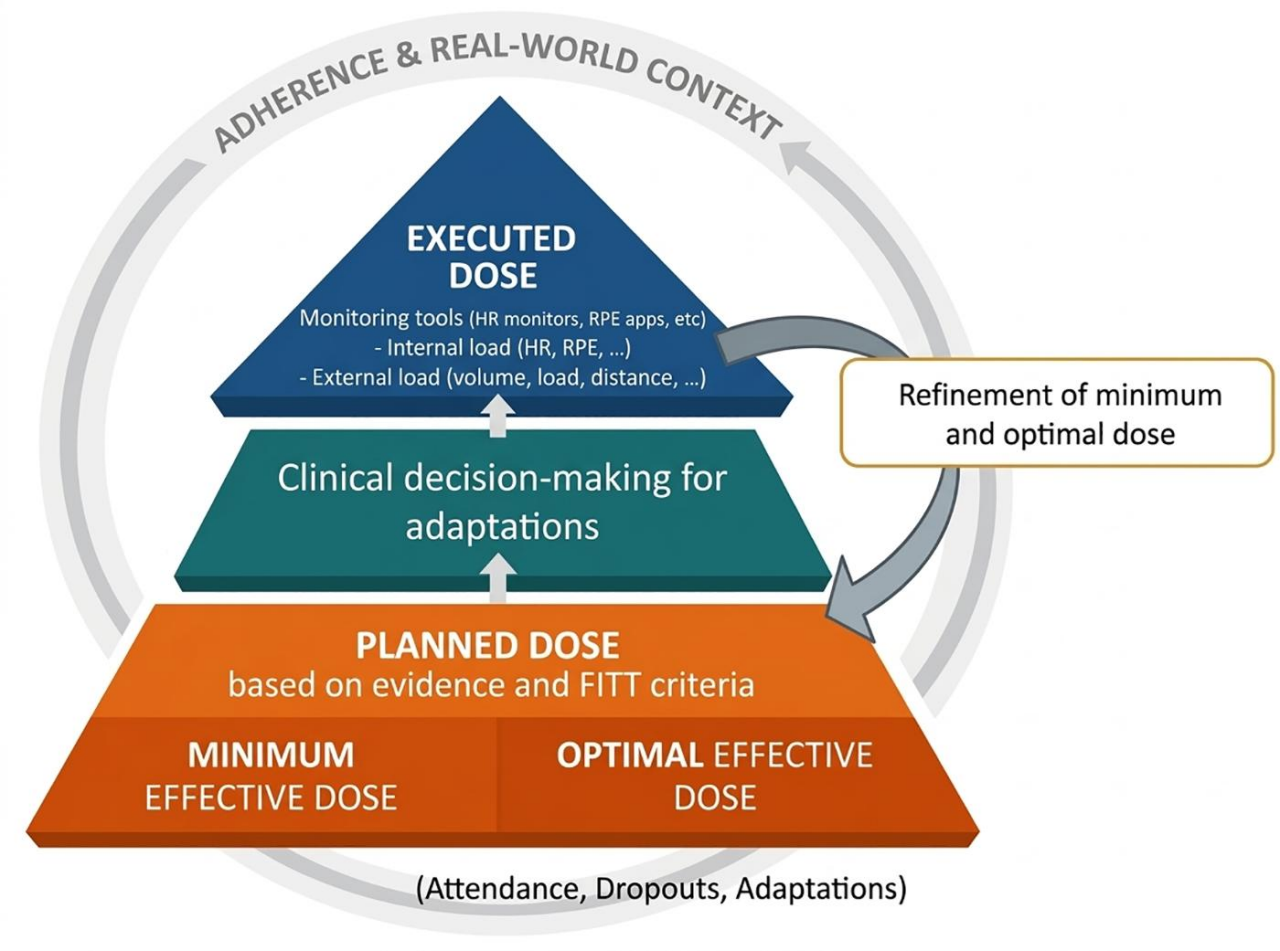
Exercise dose as a dynamic, multilevel process. The framework highlights four key areas for improving interpretability, evidence quality, and clinical translation, from planned to executed dose in real-world settings.

To establish exercise as a true therapeutic intervention in fibromyalgia, intended adherence to FITT principles alone is insufficient. Given the disease's fluctuating nature and the need for symptom-driven adaptations, exercise dose must be precisely defined, actively monitored, and transparently reported based on what is actually executed, including internal and external load, session-level modifications, and adherence over time. In this Opinion article, we outline four critical areas requiring improvement arguing that fibromyalgia research must move beyond “prescribing a program” toward the deliberate administration of exercise as a therapeutic dose. These concepts are summarized in Figure 2, which illustrates exercise dose as a dynamic, multilevel process—from planned to executed dose within a real-world clinical context. Each level of this framework is discussed in detail in the following sections.

## 2 Discussion

### 2.1 Standardizing the reporting of planned exercise dose

Niu et al. (2) reported that 10 of 22 exercise interventions showed low or uncertain compliance with ACSM recommendations, largely due to insufficient reporting of key FITT parameters (alongside discrepancies with recommended exercise doses), thereby limiting meaningful interpretation of dose–response relationships. To address poor reporting, other recent reviews (13) relied on the Adult Compendium of Physical Activities to classify exercise modalities using metabolic equivalent (MET) values. Although useful for large-scale epidemiological comparisons, MET-based classifications assume fixed metabolic costs and fail to capture the variability inherent to real-world exercise prescription. For instance, resistance training described as “multiple exercises, 8–15 repetitions at varied resistance” may be assigned a single MET value despite encompassing intensities ranging from very light to near-maximal effort, underscoring the limitations of METs for precisely characterizing exercise dose in clinical trials.

Accurate and clinically meaningful reporting therefore requires structured descriptions of FITT principles. Mind–body exercise modalities commonly used in fibromyalgia, such as yoga or Tai Chi, are defined as practices integrating physical movement with mental focus and breath regulation. In this context, the FITT framework should be expanded to ‘FITT-M’ to incorporate the Mindfulness (or mental) component—including attentional focus, breathing control, and mind–body integration—as a core dimension of the therapeutic dose. Although this component is inherently more difficult to quantify than traditional FITT parameters, its inclusion is essential to more accurately reflect the multidimensional nature of these interventions. This is further supported by recent evidence indicating that mind–body exercise therapies are a beneficial adjunctive approach for improving a range of symptoms in fibromyalgia (14).

The Consensus on Exercise Reporting Template (CERT) (15) provides a 16-item checklist covering key domains of exercise delivery and is strongly recommended to improve reporting completeness, reproducibility, and clinical translation (16). Complementary frameworks, such as PRIRES for resistance exercise interventions (17), may provide additional modality-specific guidance alongside CERT. Table 1 summarizes the CERT checklist and incorporates additional considerations proposed in this work to enhance transparency in the reporting of FITT criteria, clinical decision-making, and monitoring and adherence strategies. Adoption of CERT—particularly through explicit specification of planned FITT components in item 13—would allow a more precise characterization of exercise prescriptions, moving beyond generic MET-based classifications. Future systematic and methodological reviews should quantify the proportion of fibromyalgia trials complying with individual CERT items to better characterize current reporting gaps.

**Table 1:** Consensus on Exercise Reporting Template (CERT)–based checklist and reporting considerations for exercise interventions in fibromyalgia.

Section/ topic	Item	Checklist item	Expanded information	Rationale
WHAT: materials	1	Detailed description of the type of exercise equipment		
WHO: provider	2	Detailed description of the qualifications, expertise and/or training		
HOW: delivery	3	Describe whether exercises are performed individually or in a group		
	4	Describe whether exercises are supervised or unsupervised; how they are delivered		
	5	Detailed description of how adherence^a^ to exercise is measured and reported	• **Operational definition of adherence:** e.g., attendance to prescribed exercise sessions • **Evaluate baseline kinesiophobia** [e.g., Tampa Scale of Kinesiophobia-11 (TSK-11)]. • **Adherence indicator:** Session attendance (planned vs. completed sessions) • **Documentation of non-adherence:** Reasons for non-attendance (e.g., symptom-related, logistical). Method of data collection (attendance logs, brief self-report). • **Adherence monitoring:** Tools used to record attendance. Frequency and responsibility for data collection • **Behavior-change framework:** Theoretical model underpinning adherence support (e.g., Self-Determination Theory). Planned techniques to promote attendance (e.g., goal setting, feedback, autonomy support)	Systematic reporting of exercise adherence is essential for accurate dose interpretation and intervention validity.
	6	Detailed description of motivation strategies		
	7a	Detailed description of the decision rule(s) for determining exercise progression	• **Triggers for progression or modification** ○ Exercise-related indicators (e.g., achievement of target repetitions, reduced perceived effort at a given workload, improved technical execution, planned progression increasing intensity/volume, etc.) ○ Symptom-related indicators (e.g., pain, fatigue, flare-ups, sleep disturbance). • **Decision thresholds:** Predefined criteria guiding progression or regression ○ E.g.: RPE below target range for ≥2 consecutive sessions, Ability to complete prescribed workload with correct technique, Symptom stability across sessions, 10% volume every 2 weeks… • **Type of progression or modification:** ○ Planned actions: Increase dose (e.g., intensity, volume, complexity), maintain dose, reduce dose or temporarily pause. ○ Timeframe of decision-making: Single-session/multi-session criteria for change • **Predefined flare-up management strategies to allow temporary dose adjustments and maintain participation.** • **Decision authority:** Who applies the rules (clinician, exercise professional, shared)	Reporting exercise progression decision rules makes clinical reasoning for progression or regression explicit and reproducible, supporting interpretability and replication.
	7b	Detailed description of how the exercise program was progressed		
	8	Detailed description of each exercise to enable replication		
	9	Detailed description of any home program component		
	10	Describe whether there are any non-exercise components		
	11	Describe the type and number of adverse events that occur during exercise		
WHERE: location	12	Describe the setting in which the exercises are performed		
WHEN, HOW MUCH: dosage	13	Detailed description of the exercise intervention	• **Planned FITT components** ○ Frequency (sessions per week) ○ Intensity (relative and/or absolute criteria; e.g., RPE, %HRmax, %1RM) ○ Time (session duration and total program length) ○ Type (exercise modality and main components) ○ For mind-body modalities, include the Mindfulness component (FITT-M) to specify mental engagement and breathing techniques • **Progression structure:** Planned progression of FITT components across the intervention linked to predefined decision rules (Item 7a)	Explicit description of FITT components is essential to define exercise dose, ensure reproducibility, and interpret outcomes.
	14a	Describe whether the exercises are generic (one size fits all) or tailored		
	14b	Detailed description of how exercises are tailored to the individual	• **Domains used for tailoring** ○ Clinical and symptom-related factors (e.g., pain, fatigue, flare-ups) ○ Physical capacity and prior exercise tolerance ○ Patient preferences and contextual constraints • **Tailoring variables:** FITT components subject to individual adjustment • **Decision rules for tailoring** ○ Predefined criteria guiding individual adaptations ○ Link to symptom thresholds and progression rules (Item 7a) • **Frequency of re-tailoring:** e.g., per session, weekly, predefined checkpoints. • **Decision-making:** Who tailors the intervention (clinician, exercise professional, shared)	Reporting exercise tailoring clarifies individual adaptations and supports interpretability and clinical applicability.
	15	Describe the decision rule for determining the starting level		
HOW WELL: planned, actual	16a	Describe how adherence or fidelity is assessed/measured		
	16b	Describe the extent to which the intervention was delivered as planned	• **Extent of delivery as planned:** % sessions fully as planned, partially modified, or discontinued. • **Dimensions modified.** FITT components affected by deviations • **Magnitude and duration of deviations** (temporary or sustained) • **Clinical rationale for deviations.** Reasons for modifications (e.g., symptom exacerbation, limited tolerance, contextual constraints) • **Documentation method.** Session-level records used to capture deviations and adaptations. Intended frequency and level of monitoring. • **Detailed report of executed dose.** Internal and external load variables selected to track dose execution at session level. At least one internal load measure (e.g., RPE, heart rate, heart rate variability) reported at session level. Key external load variables appropriate to the exercise modality at session level (e.g., sets, reps, load in kg, distance, duration, or movement velocity). • **Clinical variables:** Pain, fatigue, and other symptom assessed before and after exercise sessions, including delayed responses, add extra value when linked to decision rules and exercise execution.	Systematically documenting deviations from the planned intervention allows differentiation between necessary clinical adaptations and poor implementation, enabling accurate interpretation of the executed exercise dose.

^a^ In this framework, adherence refers to attendance to prescribed exercise sessions, while the effective exercise dose is captured through systematic monitoring of load.

Beyond its role as a reporting tool, applying CERT principles prospectively during exercise protocol design may further improve intervention fidelity and clinical interpretability by requiring explicit definition of key intervention components before implementation. In line with this perspective, the framework proposed here (Table 1) emphasizes the use of CERT as a guiding tool during the design and planning stages, focusing on the key domains.

### 2.2 Clinical decision-making in exercise dosing

A common limitation in exercise trials is the discrepancy between prescribed and executed exercise dose. In the al-Ándalus randomized controlled trial (*n* = 244), participants with fibromyalgia generally trained at lower heart rate intensities than those prescribed (18). This finding illustrates a recurrent scenario in both fibromyalgia research and clinical practice: even when exercise prescriptions are well defined, the delivered dose frequently diverges as a result of real-time session adjustments and clinical decision-making.

Although highly relevant, this gap is rarely documented in sufficient detail. Most trials focus on the intended intervention, providing limited insight into how and why exercise dose is modified in practice or into the clinical decision-making processes underlying such modifications. In fibromyalgia, where symptom fluctuations and variable tolerance are inherent, these decisions are central to the safe and effective delivery of exercise. Failure to report the rationale for adaptations to disease-related variability compromises the interpretation of dose–response relationships and limits opportunities to inform and improve clinical practice. Moreover, systematically identifying the factors driving dose modifications and documenting how these adaptations are implemented would enhance both mechanistic understanding and clinical applicability.

Selected CERT items can be expanded to capture this clinical decision-making process (Table 1). Item 7a allows reporting of predefined decision rules guiding progression or dose modification, item 14b documents ongoing individual tailoring, and item 16b provides a framework to report clinically justified deviations from the planned intervention. Explicit use of these items would improve transparency, clarify how exercise dose is modified in fibromyalgia trials, and enhance clinical relevance and reproducibility.

### 2.3 Executed exercise dose: the true independent variable

While CERT-based reporting provides an essential framework for describing exercise interventions, it should be complemented by systematic monitoring and detailed reporting of the exercise dose actually performed, including both external load (what is done) and internal load (how the individual responds). In conditions such as fibromyalgia, where daily symptom fluctuations often require adaptations to the prescribed program, the executed exercise dose—rather than the planned prescription—represents the true independent variable driving clinical outcomes.

Systematic monitoring of the executed exercise dose—using practical tools such as heart rate monitors and ratings of perceived exertion (RPE)–based applications—can enhance the ecological validity of exercise trials in fibromyalgia. Real-time monitoring tools—such as heart rate monitors, heart rate–based measures, RPE apps. or velocity of execution—allow accurate capture of the physiological and mechanical stimulus actually performed during each session. Real-time feedback also facilitates precise characterization of the performed dose and timely adjustment of exercise parameters, supporting individualized prescriptions and potentially reducing symptom exacerbations. Executed dose should be characterized using complementary indicators of external load (e.g., sets, repetitions, load in kg, distance, duration, or movement velocity) and internal load (e.g., RPE and heart rate–derived measures). In addition, systematic recording of symptom responses (e.g., pain, fatigue, sleep quality assessed using visual analogue scales before and after sessions) can help to more closely align training parameters and clinical decisions with health-related outcomes across the exercise program. This approach closely mirrors real-world clinical exercise practice, in which exercise is continuously adjusted based on symptom fluctuations and individual tolerance.

From a research perspective, incorporating executed-dose data would improve interpretation of dose–response relationships and strengthen future meta-analyses. As illustrated in Figure 2, systematic monitoring may also support more refined definitions of minimal and optimal exercise doses for improving health in fibromyalgia.

### 2.4 Adherence as a central determinant of exercise effectiveness

In this framework, adherence primarily refers to consistent attendance to prescribed exercise sessions, while the effective exercise dose is captured through systematic monitoring of the executed internal and external load. Adherence is a critical, yet frequently underreported, component of exercise interventions. High dropout rates and missing data—common in exercise research and particularly prevalent in fibromyalgia—compromise outcome validity and limit interpretability. Reporting only the planned or executed exercise dose, even when accompanied by descriptions of clinical decision-making, is insufficient; understanding adherence patterns and the factors that shape them is essential to accurately quantify the stimulus that is ultimately delivered.

Several barriers have been consistently associated with reduced exercise participation in fibromyalgia, including low fitness levels, reduced self-efficacy, and fear of symptom worsening (19). Screening for psychological barriers such as kinesiophobia—using tools such as the Tampa Scale for Kinesiophobia-11 [TSK-11; (20)]—may help identify patients at greater risk of poor adherence. Conversely, appropriate supervision, lower body mass index, and lower symptom severity appear to positively influence adherence (19). Together, these findings highlight that adherence is not merely a matter of attendance, but reflects complex psychological and contextual mechanisms that influence engagement with exercise over time. However, many of these key determinants, as well as others such as kinesiophobia (19), are often not assessed or reported, limiting insight into the mechanisms underlying adherence.

In this context, most trials fail to describe the behavior-change frameworks underpinning their interventions (e.g., Self-Determination Theory, Health Belief Model), despite these models being central to sustained engagement. Explicit reporting of such frameworks would clarify how adherence is promoted, strengthening reproducibility and external validity.

Adherence is inherently dynamic, shaped by symptoms, motivation, expectations, and program structure. In fibromyalgia, predefined flare-up management strategies that allow temporary adjustment of exercise dose may help maintain participation during symptom exacerbations. Detailed reporting of session attendance, reasons for non-adherence, and resulting protocol adaptations provides the most realistic estimate of the effective exercise dose. Our proposed reporting approach is summarized in item 5 of Table 1. From a clinical perspective, adherence is fundamental: even highly efficacious exercise protocols have limited real-world value if patients cannot realistically follow them. Consequently, defining an “optimal” exercise dose is only meaningful when adherence is achievable and sustained.

## 3 Conclusion

Exercise prescription in fibromyalgia must move beyond the exclusive focus on planned FITT parameters to embrace a more comprehensive and clinically grounded understanding of exercise dose. Across this Opinion, we highlight that meaningful interpretation of exercise effects depends on transparent reporting of planned prescription, explicit documentation of clinical decision-making, systematic monitoring of the executed dose, and rigorous assessment of adherence. Recognizing exercise dose as a dynamic construct—shaped by symptom fluctuations, individual responses, and real-world adaptations—is essential to strengthen dose–response interpretation, improve reproducibility, and enhance clinical translation. Ultimately, advancing exercise research and practice in fibromyalgia requires frameworks that capture not only what is prescribed, but what is actually delivered, tolerated, and sustained by patients.
